# Evaluation of inter-observer variability of bladder boundary delineation on cone-beam CT

**DOI:** 10.1186/1748-717X-8-185

**Published:** 2013-07-23

**Authors:** Kentaro Nishioka, Shinichi Shimizu, Rumiko Kinoshita, Tetsuya Inoue, Shunsuke Onodera, Koichi Yasuda, Keiichi Harada, Yukiko Nishikawa, Rikiya Onimaru, Hiroki Shirato

**Affiliations:** 1 Department of Radiation Medicine, Hokkaido University School of Medicine, Sapporo, Japan

**Keywords:** Bladder cancer, Image guided radiotherapy, Cone beam CT, Inter-observer variability

## Abstract

**Background:**

In-room cone-beam computerized tomography (CBCT) imaging is a promising method to reduce setup errors, especially in organs such as the bladder that often have large intrafractional variations due to organ movement. CBCT image quality is limited by low contrast and imaging artifacts, but few data have been reported about inter-observer variability of bladder boundary delineation on CBCT. The aim of this work was to analyze and evaluate the inter-observer contouring uncertainties of bladder boundary delineation on CBCT images in a prospective fashion.

**Methods:**

Five radiation oncologists contoured 10 bladders using the CBCT datasets of consecutive 10 patients (including 4 females) who were irradiated to the pelvic region. Prostates were also contoured in male patients. Patients who had had prostatectomy were excluded. The coefficient of variation (COV), conformity index (CI_gen_), and coordinates of center-of-mass (COM) of the bladder and prostate were calculated for each patient.

**Results:**

The mean COV for the bladder and prostate was 0.08 and 0.20, respectively. The mean CI_gen_ of the bladder and prostate was 0.81 and 0.66, respectively. The root mean square (RMS) of the inter-observer standard deviation (σ) of the COM displacement in the left-right (LR) and anterior-posterior (AP) direction was 0.79, 0.87 and 0.54 for the bladder and 0.63, 0.99 and 1.72 for the prostate. Regarding the mean COV and CI_gen_ for the bladder, the differences between males and females were not significant.

**Conclusions:**

Inter-observer variability for bladder delineation on CBCT images was substantially small regardless of gender. We believe that our results support the applicability of CBCT in adaptive radiotherapy for bladder cancer.

## Background

The bladder continually changes volume and position on a daily basis, and as a result, treating a bladder typically requires at least a 1.5- to 2-cm isotropic setup margin in radiotherapy [1,2]. Such a large margin and treatment field may result in late bladder and bowel toxicity [3,4]. Conformal irradiation of the bladder may reduce these complication risks.

Recently, various kinds of image-guidance technology, such as implanted fiducial markers, on-board kilovoltage cone-beam computed tomography (CBCT), and ultrasonograpy, are widely used [5,6]. We had previously reported the efficacy of implanted fiducial markers in reducing uncertainty due to setup error and internal organ motion [7,8], but implantation is an invasive procedure, and fiducial markers are themselves surrogates for implanted organ position and provide no information on organ deformation or volume.

Of the other image-guidance technologies, CBCT is less invasive and the most common image-guided radiation therapy (IGRT) method, providing the volumetric-anatomic information and the opportunity to localize target volumes in a few minutes before each treatment fraction. Daily online adaptive radiotherapy using pre-planned treatment plans and CBCT has received much attention for its ability to reduce setup error and the required margins, thereby reducing the dose to the bowel in external beam radiotherapy for bladder cancer [9-13]. However, CBCT images have been qualitatively described as inferior to those of diagnostic CT, which may account for the uncertainty in delineating organ boundaries described in previous studies [14,15].

Regarding delineating bladder boundaries on planning CT images, it was reported that the inter-observer variation was relatively small [16,17], but few data are available about inter-observer variation on CBCT images. Most of the available data were reported in prostate cancer patients in a retrospective fashion, and the bladder was contoured as an organ at risk. These data could contain patient selection bias and gender bias, because some preparation protocols were applied to most of the prostate cancer patients and these patients were inevitably male. The bowel and bladder preparation protocol, such as voiding and collecting urine, defecating before treatment and endorectal balloon, is used to reduce factors of influence in interfraction motion, but these procedure may affect the delineation of the bladder on CBCT images. Moreover, the effect of organs peculiar to women (e.g., uterus and ovaries) in detecting organ boundaries with CBCT images was not considered.

To study image-guided radiotherapy for bladder cancer using CBCT, we conducted a prospective contouring protocol to analyze and evaluate the inter-observer contouring uncertainties of bladder boundary delineation on CBCT images with minimal preparation. We also analyzed the inter-observer contouring uncertainties of the prostate as the benchmark to link with previously published studies.

## Methods

### Patients’ and observers’ characteristics

Since April 2011, ten consecutive patients who were irradiated to the pelvic region were enrolled in this multiple-observer contouring study. The ethical committee of Hokkaido University Hospital approved this study (number 010-0305). Patients who had had prostatectomy were excluded. The individual patients’ characteristics are listed in Table 1. Of the five patients with bladder tumors, two patients received ureteral stents prior to radiotherapy. Fiducial markers were not placed in any of the patients.

**Table 1:** Patient characteristics

**Patient**	**Age**	**Gender**	**Tumor site**
A	90	Female	Bladder
B	70	Female	Uterus
C	83	Female	Bladder
D	71	Female	Bladder
E	68	Male	Prostate
F	83	Male	Bladder
G	90	Male	Bladder
H	77	Male	Prostate
I	69	Male	Prostate
J	74	Male	Prostate

Five physicians (four experienced radiation oncologists and one senior resident of the Department of Radiation Oncology who had worked in genito-urinary service) were recruited for the study (KN, RK, TI, SO, KY, and KH). The clinical experience of radiotherapy of all observers was ranged from 3 to 8 years with an average experience of 5.6 years.

### CBCT image acquisition

Patients with bladder cancer were asked to void just before their treatment during the treatment course, and no other bowel or bladder preparation protocol including diet-related instruction was offered to any of the 10 patients. All CBCT datasets were acquired weekly in the supine position, immediately after initial setup to skin marks. CBCT images were not used to adjust the patient’s position in this study period.

All patients were imaged and treated on a Varian Clinac iX Linear Accelerator (Varian Medical Systems, Palo Alto, CA, USA) using the kV imaging system. The CBCT images were acquired using standard factory settings of 125 kVp, 80 mA, and 20 ms per projection with a half bow-tie filter. Images were reconstructed at an axial slice thickness of 0.25 cm.

### Contouring protocol

For delineation of the organ boundaries, we used the first CBCT dataset of each patient that contained the entire bladder and prostate during the treatment course.

All observers were asked to delineate the outer contour of the whole bladder and prostate without margin for microscopic extension and seminal vesicles. In all cases the bladder was contoured as a solid organ. Contouring was performed in a blinded fashion, i.e., each observer could use only one image dataset of the patient at the time of delineation. Access to the structures drawn by other participants or the other imaging modalities (e.g., treatment planning CT, diagnostic CT, or MRI) as well as the help of a radiologist was not permitted. Contouring was carried out in the treatment planning system (Eclipse ver. 8.9, Varian Medical Systems, Inc.) using the standard tools available. Observers were free to modify window range and level of the images as preferred, and interpolation of the contours between slices was allowed. Intra-observer error was not investigated as part of this study.

### Inter-observer variation analysis

The total encompassing delineated volume and the overlapping volume between the observers’ contours were calculated using the Eclipse planning system Boolean function.

To assess inter-observer variations in organ volumes, we calculated coefficients of variation (COV = standard deviation/mean volume) for the bladder and prostate. The COVs of all observers’ contours per patient were calculated and averaged over all patients.

To evaluate the inter-observer concordance, the generalized conformity index (CI_gen_), defined as the ratio of the sum of all overlapping volumes between pairs of observers and the sum of all overlapping and all non-overlapping volumes between the same pairs [18], was used, as follows:

$$\text{CIgen}=\frac{\sum_{i,j=1}^{n}\text{pairs}\left(V_{i}\cap V_{j}\right)}{\sum_{i,j=i}^{n}\mathrm{pairs}\left(V_{i}\cup V_{j}\right)},$$

A CI_gen_ of 1 indicates 100% concordance for the volume segmentation, a CI_gen_ of 0.5 indicates 50% agreement between observers for the encompassing volume, a CI_gen_ of 0 indicates no concordance in delineation. The CI_gen_s were calculated per patient and averaged over all patients.

Coordinates of the center-of-mass (COM) of each structure in 3D were also extracted. COM displacement values along the left-right (LR), anterior-posterior (AP), and cranial-caudal (CC) direction were analyzed. As the overall mean of standard deviation, the root mean square (RMS) of the total COM standard deviation (σ) on CBCT was calculated, as follows:

$$\sigma =\sqrt{\frac{\sigma_{1}^{2}+\sigma_{2}^{2}\cdots +\sigma_{n}^{2}}{n}},$$

where σ_*i*_ indicates the standard deviation of the COM displacement value of the structure in patient *i* drawn by the respective observer in a given direction.

To evaluate the reliability of this study, we calculated the intra-class correlation coefficients (ICC(2,*k*)), where *k* represents the number of observers. The ICC is a tool for reliability analysis, which is defined from the variance components as

$$\text{ICC}=\frac{\sigma_{\mathrm{ws}}^{2}}{\sigma_{\mathrm{ws}}^{2}+\sigma_{\mathrm{bs}}^{2}},$$

where the subscripts *ws* and *bs* denote within-subject and between-subjects variance, respectively. As the true value of the variance is unknown, we use estimates from analysis of variance (ANOVA) analysis, which provides the variance components with respective mean squares between patient cases (MS_bpat_), within one patient case (MS_wpat_), between observers (MS_obs_), and between error terms (MS_err_). As different forms of ICC are described in the literature, we selected ICC(2,k) for the situation in which some physicians (observers) of the department delineated organ boundaries in multiple patients, once for each patient. The ICC can be used to assess the overall reliability of *k* observers in contouring all *n* given cases (ICC(2,*k*)), as follows:

$$\text{ICC}\left(2,k\right)=\frac{MS_{\text{bpat}}-MS_{\text{err}}}{MS_{\text{bpat}}+\frac{MS_{\text{obs}}-MS_{\text{err}}}{n}}$$

ICC values < 0.4 indicate poor reliability, ICC values between 0.4 and 0.6 indicate moderate reliability, and ICC values > 0.6 or 0.8 denote substantial or excellent reliability, respectively [19].

Statistical analysis was performed with JMP 9.0.3 (SAS Institute, Cary, NC, USA) and SPSS 11.5 (SPSS Inc., Chicago IL). Statistical significance of the outcome was assumed for p<0.05.

## Results

**Figure 1:**
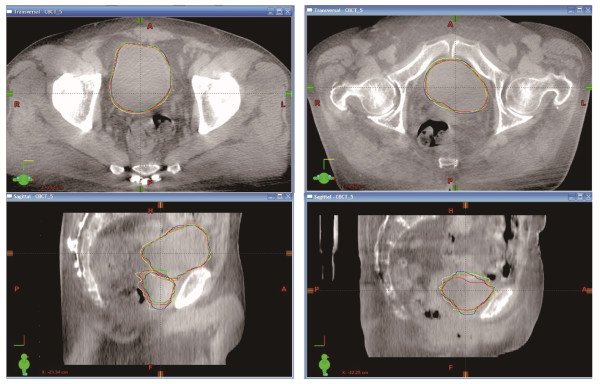
**Example of organ boundary delineation in a male and a female. **The two leftmost and two rightmost images are transaxial (upper) and sagittal (lower) images through the center of the bladder of patient H (male) and patient C (female), respectively.

All observers were able to contour both the bladder and the prostate using the CBCT images. Figure 1 shows the variation between observers for a male patient and a female patient. The mean contoured volume (range of standard deviation of the volume) of the bladder for all patients was 32.4-204.0 cm^3^ (2.1-17.2 cm^3^). For the male patient, the mean volume of the prostate was 19.6-111.9 cm^3^ (4.0-7.9 cm^3^).

The average ICC(2,*k*) values of observers for the bladder was 0.9954. When separated by gender, the average ICC(2,*k*) values for male and female bladder was 0.9980 and 0.9873, respectively. This suggests correlation between the observers in both gender. The average ICC(2,*k*) values for the prostate was 0.9950.

### COV

The mean COV (± standard error of the mean) of the bladder and prostate was 0.08 (± 0.01) and 0.20 (± 0.04), respectively. Data of individual patients are shown in Table 2. The difference of COV between the bladder and prostate was statistically significant (p=0.0442). Regarding the mean bladder COV between the male patient and the female patient, the difference was not significant (0.07 for the male, 0.08 for the female, p=0.7745).

**Table 2:** Patient-specific results of volumes and COV

**Patient**	**Gender**	**Bladder volume (cm**^**3**^**)**			**COV**	**Prostate volume (cm**^**3**^**)**			**COV**
		**Mean**	**Range**	**SD**		**Mean**	**Range**	**SD**	
A	Female	103.2	94.4-111.4	6.5	0.06	-	-	-	-
B	Female	71.3	68.5-74.2	2.1	0.03	-	-	-	-
C	Female	82.7	67.7-96.4	10.6	0.13	-	-	-	-
D	Female	166.1	147.4-193.2	17.2	0.10	-	-	-	-
E	Male	83.4	76.0-87.8	5.2	0.06	20.7	14.3-32.2	6.8	0.33
F	Male	46.5	43.7-48.9	2.1	0.05	25.6	16.5-37.9	7.9	0.31
G	Male	204.0	195.4-222.6	10.9	0.05	19.6	14.1-25.8	4.2	0.21
H	Male	123.2	120.6-129.7	3.7	0.03	31.6	27.2-37.0	4.0	0.13
I	Male	32.4	25.2-41.6	6.0	0.18	32.9	28.8-41.5	5.3	0.16
J	Male	172.1	158.6-182.7	8.9	0.05	111.9	106.0-116.9	4.1	0.04

Abbreviations: *SD* Standard deviation, *COV* Coefficients of variation.

### CI_gen_

**Figure 2:**
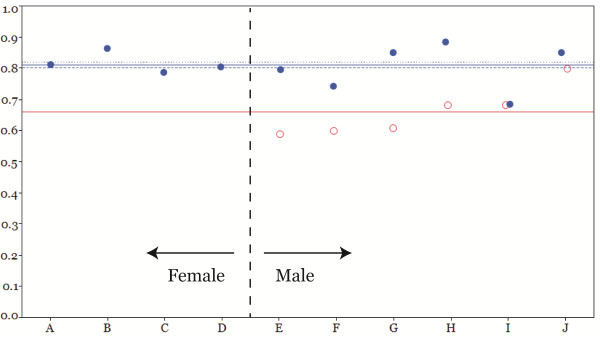
**Generalized conformity index (CI**_**gen**_**) for the study patients. **The horizontal blue solid line indicates the mean of the overall CI_gen_s of ten bladders (0.81), and the blue dotted line and blue dashed line indicate the mean of the CI_gen_s of the female bladders (0.82) and male bladders (0.80), respectively. The red solid line indicates the mean of overall CI_gen_s of prostates (0.66).

The mean CI_gen_ (± standard error of the mean) of the bladder and prostate was 0.81 (± 0.02) and 0.66 (± 0.03), respectively (Figure 2). The difference of mean CI_gen_ between the bladder and prostate was statistically significant (p=0.0038). The difference of mean bladder CI_gen_ between the male patient and the female patient was not significant (0.80 for the male, 0.82 for the female, p=0.7099).

### COM

**Figure 3:**
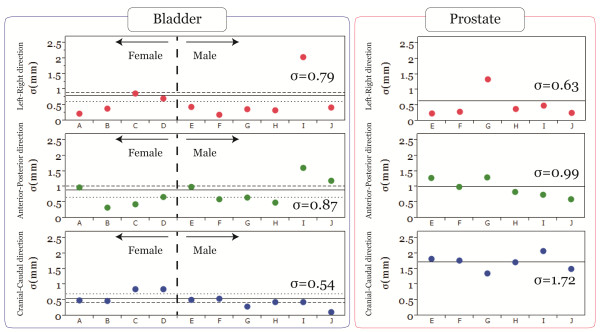
**The standard deviation (SD) of the center of mass (COM) displacement value of the structure along each direction. **The horizontal solid line indicates the root mean square (RMS) of the overall SD (σ) of the bladders and prostates. The dotted line and dashed line indicate the σ of the female bladders and male bladders, respectively.

The RMS of the standard deviation (σ) of the inter-observer COM displacement was 0.79, 0.87, and 0.54 for the bladder and 0.63, 0.99, and 1.72 for the prostate in the LR, AP, and CC direction, respectively (Figure 3). Regarding the COM location for the bladder in terms of gender, σ was 0.89, 1.00, and 0.41 for males and 0.60, 0.64, and 0.68 for females in the LR, AP, and CC direction, respectively.

## Discussion

CBCT is an established strategy for 3D image guidance during treatment. It provides reasonable soft-tissue contrast and enables the verification of both target volume and organ at risk displacements. Prostate cancer is one of the most frequently targeted tumors using CBCT, and many studies have reported its efficacy [20,21]. The authors of some of these studies reported that the accuracy of kilovoltage CBCT was similar to that of kV fiducial imaging for prostate patients with implanted gold fiducial markers [22,23], but the subjective CBCT image quality was worse compared with that of diagnostic CT or MRI [14] and large inter-observer variability in organ boundary delineation was expected.

CBCT has been found to be useful, especially in organs expected to have large intrafractional error due to organ movement, such as the bladder, but up to now few data have been available about the accuracy of bladder delineation by CBCT. Foroudi et al. reported 4 patients with bladder cancer in whom the conformity index for CBCT was not significantly inferior to that of conventional planning CT in the contouring of the whole bladder as the clinical target volume (CTV) [24]. However, most of the available data were reported in prostate cancer patients in whom the bladder was contoured as an OAR, and thus there could be some biases, such as patient selection, gender, and preparation protocol before each treatment. The aim of the present study was to analyze and evaluate the inter-observer contouring uncertainties of bladder boundary delineation on CBCT images in a prospective fashion.

There is no general consensus in the literature regarding the analysis of inter-observer variability in delineation. Recently, Fotina et al. reported common relationships between the different parameters reported and discussed the minimal set of parameters needed for “full description” of variability in delineation. They concluded that a combination of descriptive statistics, overlapping measurements, and statistical measures of agreement was required for a full reporting [19]. We selected the COV and ICC(2,*k*) as parameters of descriptive statistics and statistical measures of agreement, and the CI_gen_ as an indication of overlapping measurements as appropriate tool independent from the number of observers, following the suggestion of Kouwenhoven et al. [18].

The results of this study were in accordance with those of previous reports. Lütgendorf-Caucig et al. reported that the mean COV and CI_gen_ for the bladder on CBCT imaging was 0.06 ± 0.02 and 0.82 ± 0.05, and RMS (σ) of the COM displacement for the bladder was smaller than 1mm in all directions. While for the prostate, the mean COV and CI_gen_ was 0.24 ± 0.07 and 0.57 ± 0.09 and σ of the COM displacement was 0.4 mm (LR), 1.1 mm (AP), and 1.7 mm (CC), respectively [14]. Weiss et al. reported the patient-averaged COV was 0.08 for the bladder and 0.19 for the prostate [15]. White et al. reported the average standard deviation for COM displacements of the prostate was 0.7 mm (LR), 1.8 mm (AP), and 2.8 mm (CC) [25].

The limitation of this study is that the number of patients and observers was small especially when we separated them by gender. We could not find an apparent difference between males and females in either the mean bladder COV or the mean CI_gen_ in our analysis but it is not conclusive. Regarding the σ of COM displacement, the significance of difference between males and females could not be statistically analyzed, but σ along each direction was quite small (equal to or less than 1 mm).

## Conclusions

Inter-observer variability for bladder delineation on CBCT images was substantially small regardless of gender. We believe that our results support the applicability of CBCT in adaptive radiotherapy for bladder cancer.

## Abbreviations

CBCT: Cone-beam computed tomography; COV: Coefficients of variation; CIgen: Generalized conformity index; COM: Center-of-mass; IGRT: Image-guided radiation therapy; CT: Computed tomography; MRI: Magnetic resonance imaging; RMS: Root mean square; ICC: Intra-class correlation coefficients.

## Competing interests

The authors declare that they have no competing interests.

## Authors’ contributions

KN conceived of the study, participated in the design of the study, carried out the treatment planning, participated in data collection and interpretation and in drafting and final revising of the manuscript, and performed the statistical analysis. TI, SO, KY, and KH carried out the treatment planning, participated in data interpretation and in drafting and final revising of the manuscript. RK and YN participated in data collection and interpretation and in drafting the manuscript. SS and RO participated in the design of the study and in drafting and final revising of the manuscript. HS participated in study design and coordination and in drafting and final revising of the manuscript. All authors read and approved the final manuscript.
